# Exposure of *Paracoccidioides brasiliensis* to Mebendazole Leads to Inhibition of Fungal Energy Production

**DOI:** 10.3390/antibiotics12020206

**Published:** 2023-01-18

**Authors:** Olivia Basso Rocha, Kleber Santiago Freitas e Silva, Dayane Moraes, Clayton Luiz Borges, Célia Maria de Almeida Soares, Maristela Pereira

**Affiliations:** Laboratory of Molecular Biology, Institute of Biological Sciences, Federal University of Goiás, Goiânia 74690-900, Brazil

**Keywords:** paracoccidioidomycosis, repositioning, proteomics

## Abstract

Paracoccidioidomycosis (PCM) is a fungal disease caused by organisms of the genus *Paracoccidioides* spp. The treatment of the disease is lengthy and includes several adverse effects. Various methodologies focus on the search for new treatments against fungal disease, including the repositioning of drugs. Our group showed the fungicidal effect of mebendazole in *P. brasiliensis* cells. Thus, understanding the effect of exposing fungal cells to mebendazole is significant for further studies in order to demonstrate it as a potential drug for the treatment of PCM. A proteomic analysis of *P. brasiliensis* exposed to mebendazole was carried out. Analyses showed that exposure strongly affected the pathways related to energy production, such as glycolysis, fermentation, and the electron transport chain. The quantification of adenosine triphosphate (ATP) and mitochondrial activity demonstrated that the drug alters the electron chain, resulting in an increase in oxidative stress. Enzymes such as superoxide dismutase (SOD) and cytochrome c oxidase (Cyt C) were repressed in cells exposed to mebendazole. The concentration of ethanol produced by the cells under treatment demonstrated that the attempt to produce energy through fermentation is also arrested. Thus, the drug inhibits fungal growth through changes in energy metabolism, making it a promising compound for use in the treatment of PCM.

## 1. Introduction

Fungal mycoses caused by thermodimorphic pathogens have been increasing over the last decade and among these, we highlight paracoccidioidomycosis (PCM), whose pathogen is a fungi of the genus *Paracoccidioides* spp. [1]. The species of the genus are found in the soil in the form of mycelia. Upon contact with the host, the temperature shift induces the transition of the pathogen into the yeast form during infection [2]. PCM is an endemic disease in Latin America that mainly affects individuals who work in rural areas and who participate in soil manipulation. The lungs are the organs most affected by the infection, but the disease can cause damage to the mucous membranes and spread to other organs, possibly even reaching the nervous system [3].

The treatment of the disease is lengthy, lasting up to 2 years, causing several adverse effects, such as hepatotoxic consequences, headaches, and nausea. These aspects often lead patients to give up treatment [4]. Withdrawal from treatment leads to several sequelae, or even death. Thus, seeking new treatments that reduce these adverse effects or the duration of treatment is of paramount importance for individuals infected with PCM. Several approaches have been used to search for new therapies against systemic mycosis, including in silico methodologies, natural compounds assays, the synthesis of new compounds with specific targets, and the repositioning of drugs [5].

Mebendazole [(5-benzoyl-3H-benzoimidazol-2-yl)amino] is a benzimidazole with an anthelmintic activity, widely used in the treatment of intestinal parasitic diseases [6]. The antifungal effect of the drug has already been observed in *C. neoformans*, directly affecting the biofilm formation capacity. In addition, its anticryptococcal effect was similar to that of amphotericin B (AmB) [7]. Mebendazole is a broad-spectrum anthelmintic that has been used in humans for a long period of time. It is a safe drug, with no significant side effects when used at recommended doses [8].

Our group developed a computational drug repositioning approach, using chemogenomics to search for drugs already approved or being evaluated in clinical trials, that could be used as antifungals against PCM. This approach is based on analyzing possible interactions between those drugs and specific target proteins of *Paracoccidioides* spp. Among the drugs tested, mebendazole stood out for its minimum inhibitory concentration (MIC) value, which ranged from 3.3 to 26.4 µM [9].

The antiparasitic mechanism of action of mebendazole is already known, and it involves the inhibition of the polymerization of the tubulins in helminths, preventing the growth of parasites [10]. The mechanism of action that causes the antifungal effect of the drug is still unknown. Thus, the objective of the present research is to understand, through a proteomic analysis, which proteins are differentially regulated in *Paracoccidiodies brasiliensis* during exposure to mebendazole and to make possible inferences about its antifungal mode of action against this pathogenic fungus.

## 2. Results

### 2.1. Viability Is Decreased and Growth Reduced in Cells Exposed to Mebendazole

**Figure 1:**
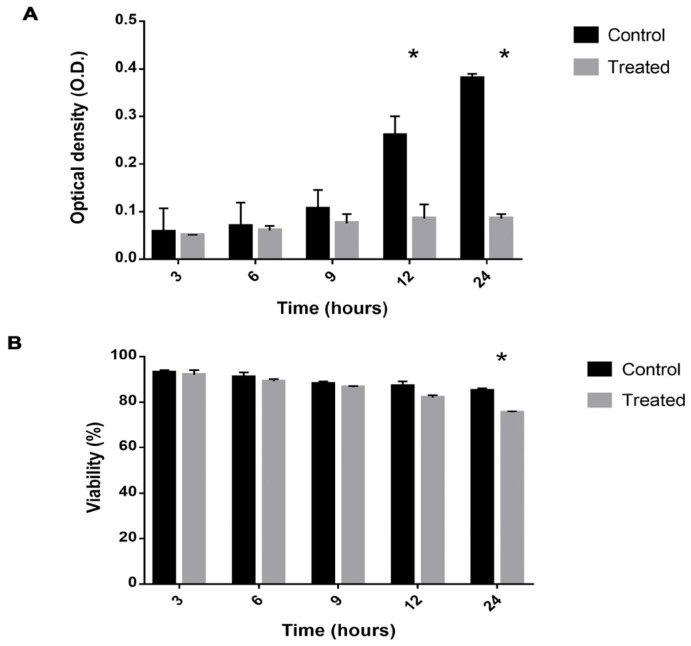
Viability and growth of *P. brasiliensis* cells exposed to mebendazole. (**A**) Viability of *P. brasiliensis* cells verified by counting using trypan blue. (**B**) Cell growth verified by optical density. The * represents a significant difference between the samples (*p* ≤ 0.05). The error bar represents the deviation between the biological triplicates.

The viability and growth of *P. brasiliensis* cells after exposure to mebendazole were evaluated in order to verify the best point for protein extraction and to identify the proteome profile of cells treated with mebendazole (Figure 1). Growth was inhibited after 12 h of exposure to the compound, where growth was significantly lower in the treated compared to the control samples. Cell viability was affected after 24 h of exposure, being significantly lower in the treated samples. At 12 h of exposure, viability was approximately 87%, but growth was already being affected. Hence, this was the point chosen for the proteomic analysis.

### 2.2. Exposure to Mebendazole Alters the Energy Metabolism of Cells

**Figure 2:**
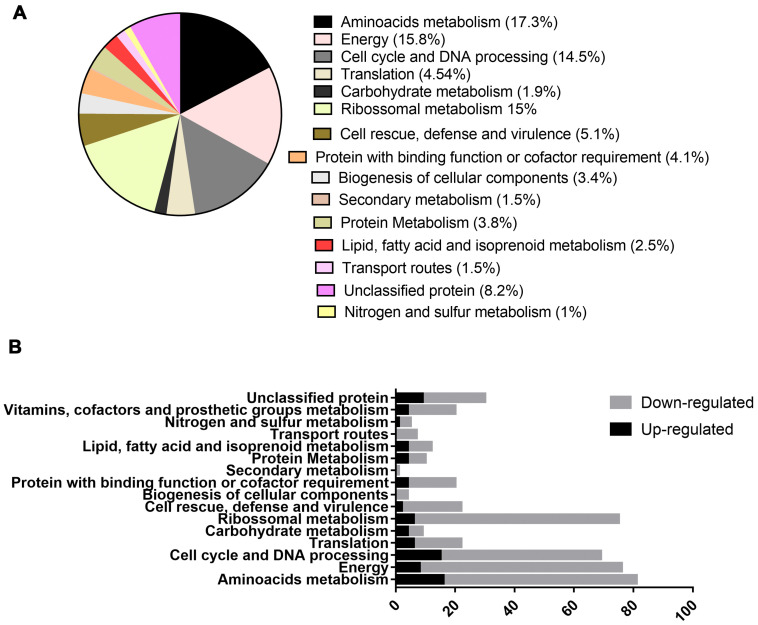
Functional characterization of regulated proteins in *P. brasiliensis* after exposure to mebendazole. (**A**) Percentage of regulated protein classes identified by proteomic analysis after exposure to mebendazole, (**B**) comparison of up- and downregulated proteins within the enriched functional groups.

Proteomic analysis identified 462 proteins, of which 136 were upregulated (Supplementary Table S1) and 326 were downregulated (Supplementary Table S2). All the proteins categorized as differentially expressed had a fold change at least equal to 1.5. Functional enrichment made it possible to identify the functions of these proteins, although 38 of them could not be reannotated. The main metabolic classes of the differentially expressed proteins identified were amino acid metabolism, energy metabolism, ribosomal metabolism, and proteins related to the cell cycle and DNA processing (Figure 2A,B).

In amino acid metabolism, 80 proteins were categorized, of which 53 were downregulated and 27 were upregulated. In metabolism related to energy production, 73 proteins were identified, 14 of which were upregulated and 59 were downregulated. Regarding the proteins related to the cell cycle and DNA processing, 24 were upregulated and 43 were downregulated, while in regards to the translation, the metabolism of most of the proteins was downregulated. Carbohydrate metabolism exhibited 9 regulated proteins, 7 upregulated and 2 downregulated. Ribosomal metabolism was one of the processes most significantly affected, with 73 proteins regulated, 55 downregulated and 18 upregulated. Proteins related to cell defense and virulence were also regulated; 6 were upregulated and 18 were downregulated.

Protein metabolism showed 18 regulated proteins, 5 upregulated and 13 downregulated. A total of 19 proteins regarding the binding function or cofactor requirements were regulated, 14 were downregulated and 5 were upregulated. Proteins related to the biogenesis of cellular compounds accounted for 12 downregulated and 3 upregulated proteins. Other types of metabolism were regulated, such as secondary metabolism, nitrogen metabolism, transport routes and degradation of fatty acid, but the number of categorized proteins was smaller, with respectively 1, 5, 7, and 12 regulated proteins.

**Figure 3:**
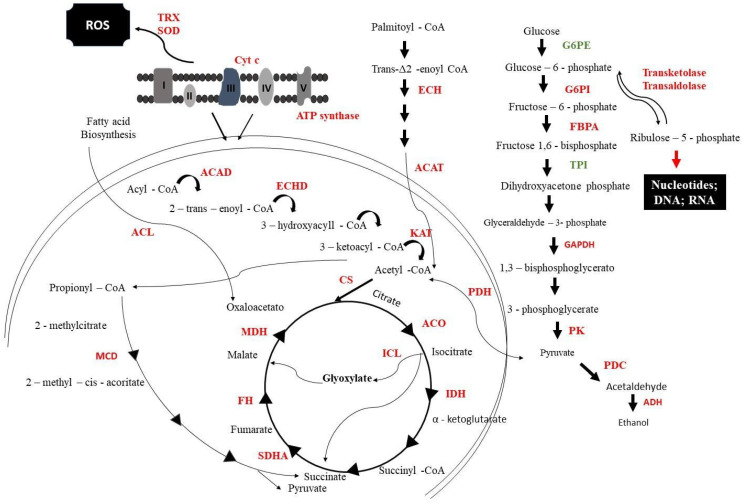
Metabolic model of *P. brasiliensis* response to mebendazole exposure. From the image, it is possible to verify enzymes that are downregulated (red) and upregulated (green). G6PE: glucose-6-phosphate; G6PI: glucose-6-phosphate isomerase; FBPA: fructose1,6-biphosphate aldolase; TPI: triosephosphate isomerase; GAPDH: glyceraldehyde 3-phosphate dehydrogenase; PK: pyruvate kinase; PDC: pyruvate decarboxylase; PC: pyruvate carboxylase; ADH: alcohol dehydrogenase; ACAD: acyl-CoA dehydrogenase; ECHD: enoyl-CoA hydratase; ACAT: cholesterol acyltransferase; KAT: ketoacyl-CoA thiolase; PDH: pyruvate dehydrogenase; CS: citrate synthase; ACO: aconitase; ICL: isocitrate lyase; IDH: isocitrate dehydrogenase; KGD: ketoglutarate dehydrogenase; SDHA: succinate dehydrogenase; FH: fumarate hydratase; MDH: malate dehydrogenase; MCD: methylcitrate dehydratase; SOD: superoxide dismutase; TRX: tyrodoxine; Cyt C: cytochrome c oxidase. I-V proteins related to electron transport chain.

The energy metabolism of cells exposed to mebendazole was drastically compromised, as most of the regulated proteins were downregulated (Figure 3). Glycolysis was repressed, as proteins such as glucose-6-phosphate (G6PE), glucose-6-phosphate isomerase (G6PI), and glyceraldehyde 3-phosphate dehydrogenase (GAPDH) were expressed in smaller amounts. Fermentation in these cells was also compromised, with enzymes such as pyruvate decarboxylase (PDC) and alcohol dehydrogenase (ADH) repressed. The electron transport chain showed the downregulation of proteins such as cytochrome c oxidase (Cyt C) and ATP synthase; this could cause an increase in oxidative stress that would be mitigated by enzymes such as thyrodoxine (TRX) and superoxide dismutase (SOD); however, these were also downregulated after exposure to the compound.

Important fungus-specific cycles, such as the methylcitrate and glyoxylate cycles, were also repressed. The methylcitrate dehydratase (MCD) that is part of the methylcitrate cycle was downregulated; this cycle indirectly produces energy as it is responsible for oxidizing propionyl-COA to pyruvate. The glyoxylate cycle was compromised, as isocitrate lyase (ICL), isocitrate dehydrogenase (IDH), and malate (MDH) were less expressed after exposure to mebendazole.

### 2.3. Mitochondrial Activity and ATP Production Is Decreased by Exposure to the Mebendazole

**Figure 4:**
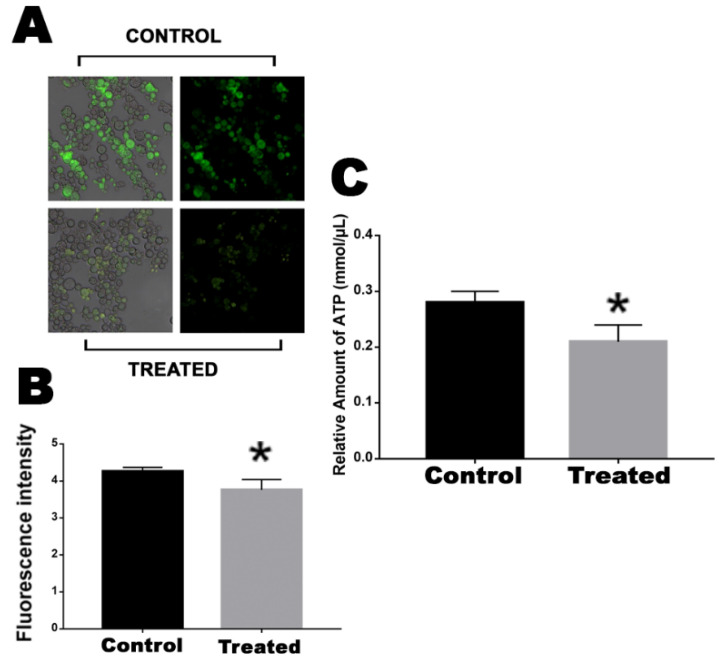
Evaluation of mitochondrial activity and ATP production in cells after exposure to the compound. (**A**) Fluorescence photomicrographs stained with MitoTracker Green to show the mitochondrial activity of cells after 12 h of exposure or no exposure to mebendazole. (**B**) Fluorescence intensity (in pixels) graph analyzed using the AxioVision V 4.8.2.0 software (Carl Zeiss, Dublin, CA, USA). (**C**) Quantification of ATP produced by cells that were exposed or not exposed to mebendazole. The * represents a significant difference between the samples (*p* ≤ 0.05). The error bar represents the deviation between the biological triplicates.

Central enzymes related to the electron transport chain, such as ATP synthase and Cyt C, were downregulated. In this way, we analyzed whether the mitochondrial activity of cells that were exposed or not to mebendazole were affected or not, through fluorescence microscopy using MitoTracker Green, and the ATP production of the cells was quantified. Cells exposed for 12 h to mebendazole showed lower fluorescence intensity than did the control cells, indicating that their mitochondrial activity was repressed (Figure 4A,B). ATP quantification showed that cells treated with the compound produced a lower concentration of ATP when compared to control cells (Figure 4C).

### 2.4. Oxidative Stress in Cells Is Increased after Incubation with Mebendazole

**Figure 5:**
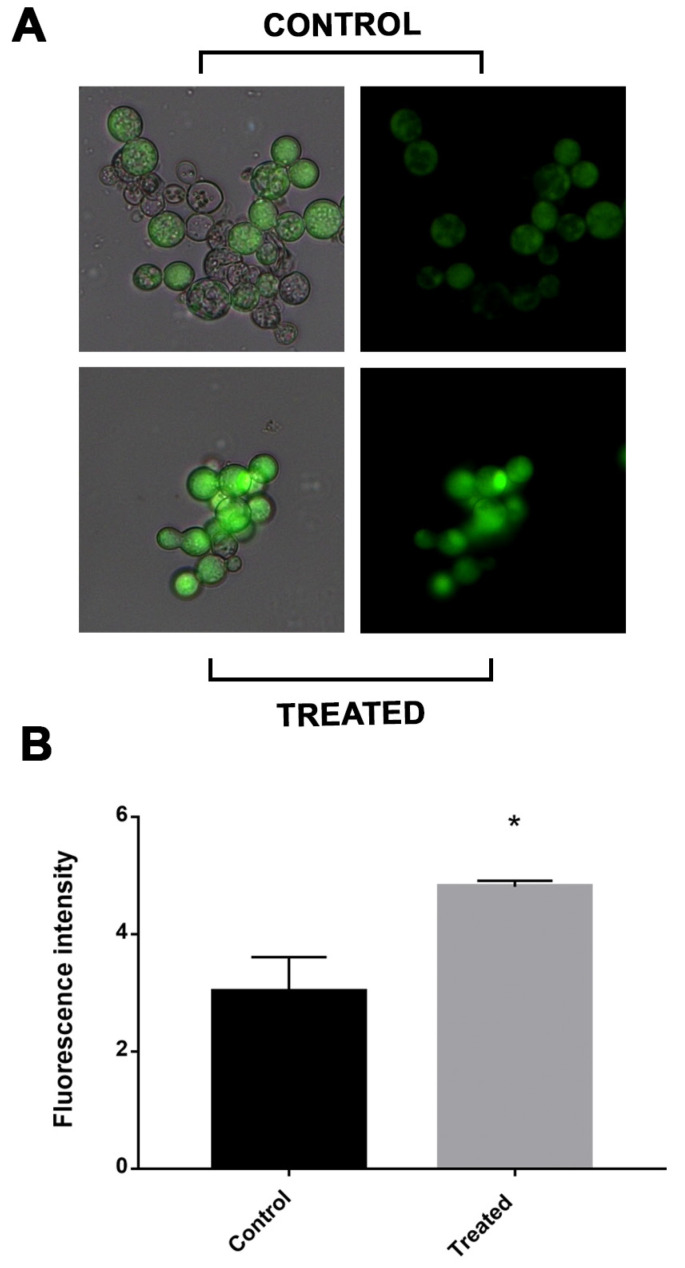
Measurement of ROS production in *P. brasiliensis* cells after exposure to mebendazole. (**A**) Fluorescence photomicrographs show cells labeled with DCFH-DC indicating ROS production after treatment with mebendazole (treated) or without treatment (control). (**B**) Fluorescence intensity (in pixels) graph analyzed using the AxioVision V 4.8.2.0 software (Carl Zeiss). The * indicates a significant difference between the control and treated samples, with a *p* value ≤ 0.05. The error bar represents the deviation between the biological triplicates.

The electron transport chain was impaired in cells exposed to mebendazole. There are reports that electron transport chain disruption would increase the intracellular concentration of ROS. In addition, the enzymes related to the electron chain, such as SOD and TRX, were downregulated. Thus, through fluorescence microscopy with cells labeled with DCFH-DC, we verified whether there was a greater oxidative stress in cells after exposure to the compound. The treated cells showed a significantly higher fluorescence intensity compared to the control cells, demonstrating that the latter were undergoing greater oxidative stress (Figure 5).

### 2.5. SOD and Cytochrome c Oxidase Enzymatic Activities Are Affected by Exposure to Mebendazole

**Figure 6:**
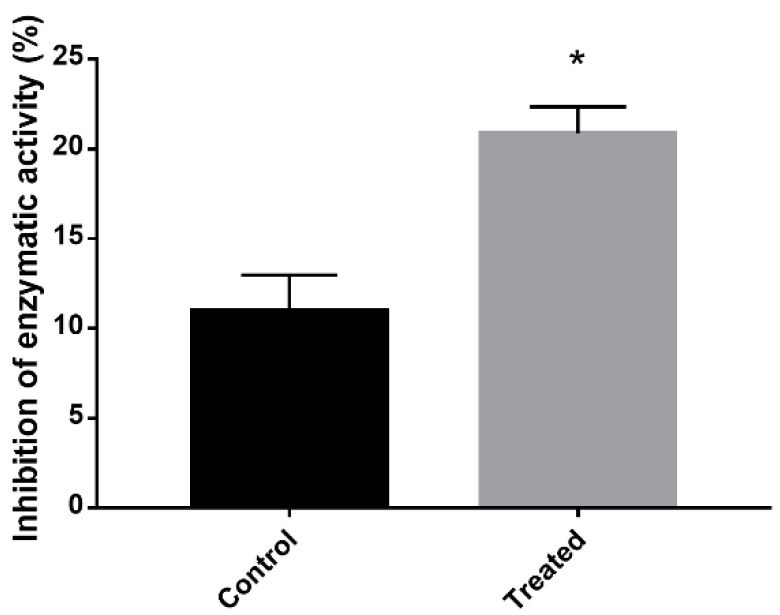
SOD enzymatic activity inhibition after exposure to mebendazole. Quantification of the level of inhibition of SOD expression in cells exposed or not exposed to mebendazole. The * indicates a significant difference between the control and treated samples, with a *p* value ≤ 0.05. The error bar represents the deviation between the biological triplicates.

The results demonstrated an inhibition of the electron transport chain and an increase in oxidative stress in cells treated with mebendazole for 12 h. To confirm this hypothesis, the percentage of SOD inhibition in cells treated or not treated with the drug were verified. The SOD inhibition rate was actually higher in the exposed cells compared to the control cells (Figure 6).

### 2.6. Energy Production by Fermentation Is Compromised after Exposure to the Mebendazole

An alternative method of producing energy when other pathways are disrupted is through fermentation. The proteomic profile showed that proteins related to fermentation, such as PDC and ADH, are downregulated,. To verify whether the fermentation was actually repressed, the concentration of ethanol in the culture medium—the ethanol that was secreted by the cells—was quantified. *P. brasiliensis* cells that were exposed to mebendazole produced a lower concentration of ethanol compared to the control cells.

## 3. Discussion

Here, we found that *P. brasiliensis* cell growth was inhibited after 12 h of exposure to mebendazole (Figure 1). Other studies have also tested this anthelmintic against fungal infections. It has been shown that in general, benzimidazole compounds inhibit *C. neoformans* growth and reduce its cellular viability [7]. In addition, the drug concentration lethality of mebendazole in human cells is nine-fold lower than that concentration for AmB [11]. Mebendazole shows inhibiting effects against *C. neoformans* sibling species, such as *C. gattii*, indicating that this chemical compound can be seen as a possible prototype for the development of antifungal drugs. The antifungal activity of mebendazole was based on the inhibition of biofilm and morphological damage in *C. neoformans* [7].

The proteomic profile of cells treated with mebendazole showed several alterations when the control and experimental samples were compared. Glycolysis was repressed in cells treated with mebendazole, guaranteed by the reduced expression of glycolytic enzymes such as G6PE, G6PI, GAPDH, and pyruvate kinase (PK) (Figure 3). The strong indication of glycolysis downregulation is the reduced expression of PK, which is a glycolysis regulation point. Glycolysis is also repressed in *Paracoccidioides* spp. cells treated with different antifungal compounds, including curcumin [12], argentilactone [13], camphene thiosemicarbazide [14], as well as other conditions, such as murine infection [15] and carbon starvation [16]. This pattern follows that of other fungal species undergoing stress conditions, such as *Aspergillus flavus* treated with antifungals [17] nystatin and acetylcandidin in *Saccharomyces cerevisiae* [18], fluconazole in *Candida albicans* [19], and during infection of *C. albicans* [20].

TCA is repressed, since its main point of regulation (IDH) is less expressed under treatment with mebendazole. The pyruvate availability is reduced, since enzymes that feed pyruvate-dependent pathways are present with reduced expression. In this group, we highlight PDH that consumes pyruvate and turn it into acetyl-CoA to run TCA and PDC, which consumes pyruvate to release acetaldehyde. The last is an important virulence factor related to infection. This indicates that *P. brasiliensis* cells fail to produce energy, which impairs cell growth and viability, as shown above (Figure 1). The TCA enzymes, aconitase (ACO) and MDH (Figure 2), were also downregulated in *Paracoccidioides* spp. cells treated with camphene thiosemicarbazide [14] and in conditions that lead cells of this species to stress, such as, hypoxia [21], zinc deprivation [22], and oxidative stress [23]. Together, these results indicate that the downregulation of TCA enzymes impair energy production processes in the fungus. In addition, these enzymes are being tested as metabolic targets for several antifungals. MDH, for example, showed reduced expression in *A. flavus* cells treated with *Curcuma longa* oil, reducing the growth of this fungal species [24].

**Figure 7:**
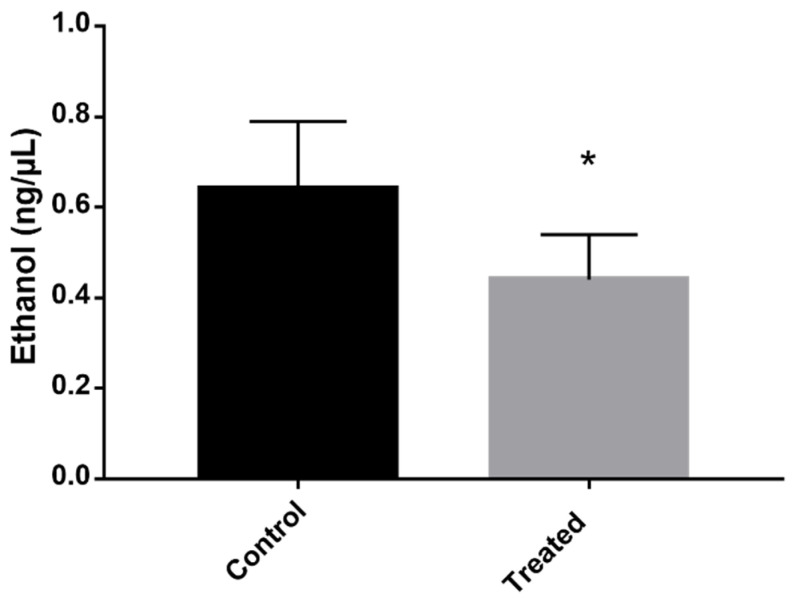
Evaluation of the concentration of ethanol produced by cells exposed to mebendazole. The production of ethanol in the cells was verified by quantifying it in the medium containing cells exposed (treated) or not exposed (control) to mebendazole for 12 h. The * indicates a significant difference between the control and treated samples, with a *p* value ≤ 0.05. The error bar represents the deviation between the biological triplicates.

Another aspect of the proteomic profile of *P. brasiliensis* cells treated with mebendazole indicates a lack of energy production. Fermentation is compromised once PDC and ADH are repressed. Interestingly, most species under stress and low levels of carbon or other nutrient sources rely on increasing fermentation levels in order to produce energy and maintain most of the active cell metabolism. This is established in *Paracoccidioides* spp. during carbon starvation [16] and murine infection [15], as well as in other fungi, such as *C. albicans* [20], *Trichoderma reesei* [25], *Aspergillus oryzae* [26], and *S. cerevisiae* [27]. Due to this discrepancy, one of the validations of the proteomic profile presented in this work was to analyze the level of ethanol produced by cells exposed to mebendazole. The results showed a reduction in the production of ethanol, confirming the proteomic results of fermentation repression (Figure 7).

To test if energy production was impaired in *P. brasiliensis* cells treated with mebendazole, we assessed the mitochondrial activity and ATP production levels via fluorescence microscopy. Cells exposed for 12 h to mebendazole exhibited lower fluorescence intensity than did the control samples, indicating that their mitochondrial activity was repressed (Figure 4), and the ATP quantification indicated a lower concentration of ATP in the experimental samples compared to that in the control. In addition, enzymes of the electron transport chain, such as ATP synthase and Cyt C, were downregulated. Respiration and energy production were also impaired in *P. lutzii* yeast cells treated with camphene thiosemicarbazide [14] in *P. brasiliensis* during murine infection [15] treated with curcumin [12]. Several prototypes of antifungals target the mitochondria and the ability of the pathogen to produce energy [28]; disrupting the oxidative phosphorylation mechanism is an efficient way to reduce fungal growth and virulence [29]. In addition, corroborating our results, ATP synthase is required for the pathogenicity of fungal species such as *C. albicans* [30] and *Mycobacterium tuberculosis* [31].

Finally, we showed that oxidative stress in cells treated with mebendazole is increased via fluorescence microscopy, and the proteomic profile in such a condition showed the downregulation of SOD, TRX, and Cyt C. The increased oxidative stress has been shown to be induced in *Paracoccidioides* spp. under several conditions, mainly during the infection of alveolar macrophages [32,33] and murine infection [15,34], treated with curcumin [12], argentilactone [13], and camphene thiosemicarbazide [14]. The mebendazole mode of action is different from that of other antifungal compounds, which generally increase the expression of enzymes related to oxidative stress. Here, we identified a downregulation of such enzymes, indicating that fungal survivability is impaired and cannot properly fight against such stress.

## 4. Materials and Methods

### 4.1. Microorganism and Culture Conditions

The cells of *Paracoccidioides brasiliensis*—Pb 18 yeasts were cultivated at 36 °C for 48 h under agitation in Fava-Netto liquid medium containing protease peptone, 1% peptone, 0.5% (*w*/*v*) meat extract, 0.5% (*w*/*v*) yeast extract, 4% glucose, 0.5% NaCl], pH 7.2. Next, 10^5^ yeast cells were incubated solely in RPMI 1640 medium with glucose (Sigma-Aldrich, St. Louis, MO, USA) or in the presence of 13.2 µM of mebendazole, as identified in our previous work, the concentration of the compound responsible for the fungicidal effects.

### 4.2. Viability and Growth Curve

The effect of cell exposure to mebendazole on viability and growth was analyzed. Viability was verified by counting using Trypan blue and a Neubauer chamber, while growth analysis was verified by reading the optical density at a wavelength of 600 nm. The two analyses were performed at the exposure times of 3, 6, 9, 12, and 24 h. These experiments were performed in triplicate. The existence of a significant difference between the control group and the treated group—the latter exposed to mebendazole—was verified by the Student’s t test, considered significant for *p* ≤ 0.05.

### 4.3. Protein Extraction

Cells were grown in RPMI 1640 medium with 13.2 µM of mebendazole for 12 h in the treatment group, while in the control group, they were cultured only in the RPMI 1640 medium. Then, samples were centrifuged at 3000× *g*, the supernatant was discarded, and the cells were resuspended in ammonium bicarbonate buffer (57 mM, pH 8.8). The cells were subjected to lysis with the addition of glass beads and 5 cycles of 30 s each in the bead beater disruptor (BioSpec Bartlesville, OK, USA). Subsequently, they were centrifuged at 12,000× *g*, and the supernatant was collected to obtain the protein extract.

### 4.4. Proteins Enzymatic Digestion

The samples were prepared for liquid chromatography using enzymatical digestion, as previously described [12]. A total of 150 µg of protein were incubated with RapiGESTTM SF (0.2%, Waters, Milford, MA, USA) at 80 °C for 15 min. We added 2.5 µL of dithiothreitol (DTT, 100 mM, GE Healthcare, Little Chalfont, UK) at 60 °C for 30 min, following the addition of 2.5 µL of iodoacetamide (300 mM, GE Healthcare, Piscataway, NJ, USA) at room temperature, and the samples were kept protected from the light for 30 min. The digestion was performed through the addition of 30 µL of a trypsin solution (0.05 μg/μL, Promega, Madison, WI, USA) and kept overnight at 37 °C. Then, 30 µL of trifluoroacetic acid (TFA, 5%) was added at 37 °C for 90 min to inactivate the digestion. The supernatant was retrieved by centrifugation at 10,000× *g* for 30 min, and then the samples were dried in a speed vacuum apparatus.

### 4.5. Liquid Chromatography and Mass Spectrometry

High-performance liquid chromatography at nanoscale (nano UPLC), coupled with tandem mass spectrometry (MSE), was used for the proteomic data acquisition, according to the methods in [9]. The pellet of each sample was resuspended in 80 µL of ammonium formate (20 mM) containing rabbit phosphorylase B (PHB, 200 fmol/µL, MassPREP™ Digestion Standard) as an internal standard. The peptides were injected into the nanoACQUITY UPLC^®^ system (Waters Corporation, Manchester, UK) and separated using a gradient of acetonitrile (11.4, 14.7, 17.4, 20.7, and 50% acetonitrile/0.1% formic acid). The mass spectra were obtained with a Synapt G1 HDMS™ mass spectrometer (Waters Micromass, Manchester, UK) equipped with a hybrid analyzer quadrupole/time-of-flight (Q-TOF). The spectrometer was programmed in “V” mode with positive ionization nanoelectrospray (nanoESI+). [Glu]1-Fibrinopeptide B human (*m*/*z* 785.8426, Sigma Aldrich, St. Louis, MO, USA) was used as a calibration solution. The samples were analyzed in triplicate.

The raw data processing was performed using ProteinLynx Global Server (PLGS) software version (3.0.2). The spectra obtained were compared with sequences from the *P. brasilensis* databases (https://www.uniprot.org/proteomes/UP000001628; accessed on 12 March 2021). The proteins were regulated if the fold-change exceeded at least the value of the 1.5 cutoff. This value is calculated by the processing performed via the PLGS software and represents the ratio of the changes between final value and the original value over the initial value.

### 4.6. Mitochondrial Activity

The mitochondrial activity of cells exposed or not to mebendazole for 12 h were verified through fluorescence microscopy using MitoTracker Green FM (Sigma-Aldrich) dye. Samples were collected and incubated for 45 m at room temperature in the dark with MitoTracker Green FM (400 µL) and then washed three times with PBS. Fluorescence analyzes were performed in triplicate and visualized using a fluorescence microscope (Zeiss Axiocam MRc-Scope A1) with a wavelength of 450–490 nm. Pixel quantification was performed with AxioVision software (Carl Zeiss), where all fluorescent and well-delimited cells were analyzed. The static difference was evaluated by the Student’s *t* test, where the difference was considered significant when *p* ≤ 0.05.

### 4.7. ATP Quantification

The colorimetric detection kit (Sigma-Aldrich) was used to quantify the ATP concentration. The reaction was performed according to the manufacturer’s instructions. The process is established by the phosphorylation of glycerol, which results in a color change detected by absorbance. For the analyses, we used 1 mL of the samples, both treated with the compound and the control. Samples were centrifuged at 3500× *g*, and the supernatant was discarded; then, the assay buffer from the kit was added to lyse and deproteinate the cells. The reading was performed at 570 nm. The analyses were performed in triplicate, and the statistical difference was evaluated by the Student’s *t* test, considered significant when *p* ≤ 0.05.

### 4.8. Reactive Oxygen Species Quantification

The amount of ROS produced in cells exposed to mebendazole for 12 h and the control samples was evaluated through fluorescence microscopy using dichlorodihydrofluorescein 2′,7′-diacetate dye (DCFH-DC). A 1 mL sample was collected and 25 µM of DCFH-DC was added, then the sample was incubated for 30 min in the dark, followed by three washes with 1X PBS. Fluorescence analyses were performed in triplicate and visualized in a fluorescence microscope (Zeiss Axiocam MRc-Scope A1) with a wavelength of 490–516 nm. Pixel quantification was performed with AxioVision software (Carl Zeiss), where all fluorescent and well-delimited cells were analyzed. The static difference was evaluated by the Student’s *t* test, considered significant when *p* ≤ 0.05.

### 4.9. Superoxide Dismutase Activity

The superoxide dismutase (SOD) activity was quantified using the SOD assay kit (Sigma-Aldrich) to determine the production of formazan dye upon reduction with O_2_^−^ by colorimetric detection via absorbance. For the analyses, 1 µg of protein extracted from cells treated with mebendazole and the control samples was used. The reading was performed in triplicate at 450 nm. The statistical difference was evaluated by the Student’s *t* test and considered significant when *p* ≤ 0.05.

### 4.10. Ethanol Quantification

The ethanol concentration was quantified by the colorimetric detection kit according to the manufacturer’s instructions (Sigma-Aldrich) The enzymatic reaction produces a color that can be measured by absorbance reading. *P. brasiliensis* cells were exposed to the mebendazole for 12 h, and the control cells were incubated under the same conditions, without mebendazole. For the analysis, 1 mL of the samples was collected and centrifuged at 3500× g. The supernatant was collected for the colorimetric evaluation. The reading was performed at a wavelength of 570 nm. Analyses were performed in triplicate, and the statistical difference was evaluated by the Student’s *t* test and considered significant when *p* ≤ 0.05.

## 5. Conclusions

The repositioning of mebendazole showed it to have antifungal potential, but its mechanism of action in *P. brasiliensis* was still unknown. This work demonstrates that a possible mechanism of action of the compound as a fungicide is its ability to interfere with the energy production of fungal cells. Important pathways involved in energy production, such as glycolysis, the electron transport chain, and fermentation, were downregulated after exposition of mebendazole. Thus, we can infer that mebendazole may be used in the future for the treatment of PCM, but in order to confirm these results with complete certainty, additional studies will be necessary.

## Data Availability

The data presented in this study are available on request from the corresponding author. The data are not publicly available because it is partially related to a subsequent publication.

## Supplementary Materials

The following supporting information can be downloaded at: https://www.mdpi.com/article/10.3390/antibiotics12020206/s1, Supplementary Table S1: *P. brasiliensis* proteins upregulated after exposure to mebendazole; Supplementary Table S2: *P. brasiliensis* proteins downregulated after exposure to mebendazole.

## References

[1] Santos L.A., Grisolia J.C., Burger E., de Araujo Paula F.B., Dias A.L.T., Malaquias L.C.C. (2020). Virulence Factors of Paracoccidioides Brasiliensis as Therapeutic Targets: A Review. Antonie Van Leeuwenhoek.

[2] Teixeira M.M., Theodoro R.C., Nino-Vega G., Bagagli E., Felipe M.S.S. (2014). Paracoccidioides Species Complex: Ecology, Phylogeny, Sexual Reproduction, and Virulence. PLoS Pathog..

[3] Bocca A.L., Amaral A.C., Teixeira M.M., Sato P.K., Shikanai-Yasuda M.A., Soares Felipe M.S. (2013). Paracoccidioidomycosis: Eco-Epidemiology, Taxonomy and Clinical and Therapeutic Issues. Future Microbiol..

[4] Shikanai-Yasuda M.A., Mendes R.P., Colombo A.L., de Queiroz-Telles F., Kono A.S.G., Paniago A.M.M., Nathan A., do Valle A.C.F., Bagagli E., Benard G. (2017). Brazilian Guidelines for the Clinical Management of Paracoccidioidomycosis. Rev. Soc. Bras. Med. Trop..

[5] Do Carmo Silva L., de Oliveira A.A., de Souza D.R., Barbosa K.L.B., Freitas e Silva K.S., Carvalho Júnior M.A.B., Rocha O.B., Lima R.M., Santos T.G., Soares C.M.d.A. (2020). Overview of Antifungal Drugs against Paracoccidioidomycosis: How Do We Start, Where Are We, and Where Are We Going?. J. Fungi.

[6] Guerini A.E., Triggiani L., Maddalo M., Bonù M.L., Frassine F., Baiguini A., Alghisi A., Tomasini D., Borghetti P., Pasinetti N. (2019). Mebendazole as a Candidate for Drug Repurposing in Oncology: An Extensive Review of Current Literature. Cancers.

[7] Joffe L.S., Schneider R., Lopes W., Azevedo R., Staats C.C., Kmetzsch L., Schrank A., Del Poeta M., Vainstein M.H., Rodrigues M.L. (2017). The Anti-Helminthic Compound Mebendazole Has Multiple Antifungal Effects against Cryptococcus Neoformans. Front. Microbiol..

[8] Chai J.-Y., Jung B.-K., Hong S.-J. (2021). Albendazole and Mebendazole as Anti-Parasitic and Anti-Cancer Agents: An Update. Korean J. Parasitol..

[9] De Oliveira A.A., Neves B.J., Silva L.d.C., Soares C.M.d.A., Andrade C.H., Pereira M. (2019). Drug Repurposing for Paracoccidioidomycosis Through a Computational Chemogenomics Framework. Front. Microbiol..

[10] MacDonald L.M., Armson A., Thompson A.R.C., Reynoldson J.A. (2004). Characterisation of Benzimidazole Binding with Recombinant Tubulin from Giardia Duodenalis, Encephalitozoon Intestinalis, and Cryptosporidium Parvum. Mol. Biochem. Parasitol..

[11] Cruz M.C., Bartlett M.S., Edlind T.D. (1994). In Vitro Susceptibility of the Opportunistic Fungus Cryptococcus Neoformans to Anthelmintic Benzimidazoles. Antimicrob. Agents Chemother..

[12] Rocha O.B., Freitas E., Silva K.S., de Carvalho Junior M.A.B., Moraes D., Alonso A., Alonso L., do Carmo Silva L., Soares C.M.A., Pereira M. (2022). Proteomic Alterations in Paracoccidioides Brasiliensis Caused by Exposure to Curcumin. J. Proteom..

[13] Prado R.S., Bailão A.M., Silva L.C., de Oliveira C.M.A., Marques M.F., Silva L.P., Silveira-Lacerda E.P., Lima A.P., Soares C.M., Pereira M. (2015). Proteomic Profile Response of Paracoccidioides Lutzii to the Antifungal Argentilactone. Front. Microbiol..

[14] E Silva K.S., da S Neto B.R., Zambuzzi-Carvalho P.F., de Oliveira C.M., Pires L.B., Kato L., Bailão A.M., Parente-Rocha J.A., Hernández O., Ochoa J.G. (2018). Response of Paracoccidioides Lutzii to the Antifungal Camphene Thiosemicarbazide Determined by Proteomic Analysis. Future Microbiol..

[15] Lacerda Pigosso L., Baeza L.C., Vieira Tomazett M., Batista Rodrigues Faleiro M., Brianezi Dignani de Moura V.M., Melo Bailão A., Borges C.L., Alves Parente Rocha J., Rocha Fernandes G., Gauthier G.M. (2017). Paracoccidioides Brasiliensis Presents Metabolic Reprogramming and Secretes a Serine Proteinase during Murine Infection. Virulence.

[16] De Sousa Lima P., Casaletti L., Bailão A.M., de Vasconcelos A.T.R., da Rocha Fernandes G., de Almeida Soares C.M. (2014). Transcriptional and Proteomic Responses to Carbon Starvation in Paracoccidioides. PLoS Negl. Trop. Dis..

[17] Delgado J., Owens R.A., Doyle S., Asensio M.A., Núñez F. (2015). Impact of the Antifungal Protein PgAFP from Penicillium Chrysogenum on the Protein Profile in Aspergillus Flavus. Appl. Microbiol. Biotechnol..

[18] Cirillo V.P., Harsch M., Lampen J.O.Y. (1964). Action of the Polyene Antibiotics Filipin, Nystatin and n-Acetylcandidin on the Yeast Cell Membrane. Microbiology.

[19] Katragkou A., Alexander E.L., Eoh H., Raheem S.K., Roilides E., Walsh T.J. (2016). Effects of Fluconazole on the Metabolomic Profile of Candida Albicans. J. Antimicrob. Chemother..

[20] Askew C., Sellam A., Epp E., Hogues H., Mullick A., Nantel A., Whiteway M. (2009). Transcriptional Regulation of Carbohydrate Metabolism in the Human Pathogen Candida Albicans. PLoS Pathog..

[21] Lima P.d.S., Chung D., Bailão A.M., Cramer R.A., Soares C.M.d.A. (2015). Characterization of the Paracoccidioides Hypoxia Response Reveals New Insights into Pathogenesis Mechanisms of This Important Human Pathogenic Fungus. PLoS Negl. Trop. Dis..

[22] Parente A.F.A., de Rezende T.C.V., de Castro K.P., Bailão A.M., Parente J.A., Borges C.L., Silva L.P., Soares C.M.d.A. (2013). A Proteomic View of the Response of Paracoccidioides Yeast Cells to Zinc Deprivation. Fungal Biol..

[23] De Arruda Grossklaus D., Bailão A.M., Vieira Rezende T.C., Borges C.L., de Oliveira M.A.P., Parente J.A., de Almeida Soares C.M. (2013). Response to Oxidative Stress in Paracoccidioides Yeast Cells as Determined by Proteomic Analysis. Microbes Infect..

[24] Hu Y., Zhang J., Kong W., Zhao G., Yang M. (2017). Mechanisms of Antifungal and Anti-Aflatoxigenic Properties of Essential Oil Derived from Turmeric (*Curcuma longa* L.) on Aspergillus Flavus. Food Chem..

[25] Chambergo F.S., Bonaccorsi E.D., Ferreira A.J.S., Ramos A.S.P., Ferreira Júnior J.R., Abrahão-Neto J., Farah J.P.S., El-Dorry H. (2002). Elucidation of the Metabolic Fate of Glucose in the Filamentous Fungus Trichoderma Reesei Using Expressed Sequence Tag (EST) Analysis and CDNA Microarrays. J. Biol. Chem..

[26] Maeda H., Sano M., Maruyama Y., Tanno T., Akao T., Totsuka Y., Endo M., Sakurada R., Yamagata Y., Machida M. (2004). Transcriptional Analysis of Genes for Energy Catabolism and Hydrolytic Enzymes in the Filamentous Fungus Aspergillus Oryzae Using CDNA Microarrays and Expressed Sequence Tags. Appl. Microbiol. Biotechnol..

[27] Johnston M., Feasting, Fasting and Fermenting (1999). Glucose Sensing in Yeast and Other Cells. Trends Genet..

[28] Li D., Calderone R. (2017). Exploiting Mitochondria as Targets for the Development of New Antifungals. Virulence.

[29] OuYang Q., Tao N., Zhang M. (2018). A Damaged Oxidative Phosphorylation Mechanism Is Involved in the Antifungal Activity of Citral against Penicillium Digitatum. Front. Microbiol..

[30] Li S., Zhao Y., Zhang Y., Zhang Y., Zhang Z., Tang C., Weng L., Chen X., Zhang G., Zhang H. (2021). The δ Subunit of F1Fo-ATP Synthase Is Required for Pathogenicity of Candida Albicans. Nat. Commun..

[31] Vestergaard M., Bald D., Ingmer H. (2022). Targeting the ATP Synthase in Bacterial and Fungal Pathogens: Beyond Mycobacterium Tuberculosis. J. Glob. Antimicrob. Resist..

[32] Chaves E.G.A., Parente-Rocha J.A., Baeza L.C., Araújo D.S., Borges C.L., de Oliveira M.A.P., Soares C.M.d.A. (2019). Proteomic Analysis of Paracoccidioides Brasiliensis During Infection of Alveolar Macrophages Primed or Not by Interferon-Gamma. Front. Microbiol..

[33] Parente-Rocha J.A., Parente A.F.A., Baeza L.C., Bonfim S.M.R.C., Hernandez O., McEwen J.G., Bailão A.M., Taborda C.P., Borges C.L., de Almeida Soares C.M. (2015). Macrophage Interaction with Paracoccidioides Brasiliensis Yeast Cells Modulates Fungal Metabolism and Generates a Response to Oxidative Stress. PLoS ONE.

[34] Castilho D.G., Navarro M.V., Chaves A.F.A., Xander P., Batista W.L. (2018). Recovery of the Paracoccidioides Brasiliensis Virulence after Animal Passage Promotes Changes in the Antioxidant Repertoire of the Fungus. FEMS Yeast Res..

