# Supplementary material for: Exposure of Paracoccidioides brasiliensis to Mebendazole Leads to Inhibition of Fungal Energy Production
Source: Antibiotics (Basel). 2023 Jan 18;12(2):206. doi: 10.3390/antibiotics12020206 (PMC9951877; doi:10.3390/antibiotics12020206)
Supplement: Supplementary file 1 [file antibiotics-12-00206-s001.zip › antibiotics-2158427-supplementary.pdf]

antibiotics

MDPI

Article

Exposure of *Paracoccidioides brasiliensis* to Mebendazole Leads to Inhibition of Fungal Energy Production

Olivia Basso Rocha *, Kleber Santiago Freitas e Silva, Dayane Moraes, Clayton Luiz Borges, Célia Maria de Almeida Soares and Maristela Pereira

Laboratory of Molecular Biology, Institute of Biological Sciences, Federal University of Goiás, Goiânia 74690-900, Brazil

* Correspondence: oliviabassorocha@gmail.com; Tel.: +55-62-3521-1110

Citation: Rocha, O.B.; e Silva, K.S.F.; Moraes, D.; Borges, C.L.; Soares, C.M.d.A.; Pereira, M. Exposure of *Paracoccidioides brasiliensis* to Mebendazole Leads to Inhibition of Fungal Energy Production. *Antibiotics* **2023**, *12*, 206. https://doi.org/10.3390/antibiotics12020206

Academic Editor: Alessia Catalano

Received: 28 December 2022
Revised: 14 January 2023
Accepted: 16 January 2023
Published: 18 January 2023

Copyright: © 2023 by the authors. Licensee MDPI, Basel, Switzerland. This article is an open access article distributed under the terms and conditions of the Creative Commons Attribution (CC BY) license (https://creativecommons.org/licenses/by/4.0/).

Table S1. ***P. brasiliensis*** **proteins up-regulated after exposure to mebendazole**

Acession number	Protein description	Fold change
	**1. Metabolism**	
	**1.1 Amino acid metabolism**	
PADG_05820	Xaa-Pro aminopeptidase	*
PADG_05193	Xaa-Pro aminopeptidase	*
PADG_00685	3-hydroxy-3-methylglutaryl coenzyme A synthase	*
PADG_06671	3-isopropylmalate dehydrogenase	*
PADG_01688	4-hydroxy-4-methyl-2-oxoglutarate aldolase/oxaloacetate decarboxylase	*
PADG_03020	Alanine--glyoxylate transaminase	*
PADG_04854	Anthranilate synthase	*
PADG_01404	Aspartate aminotransferase	*
PADG_12043	Cysteine desulfurase	*
PADG_02726	Cysteine synthase	*
PADG_08662	Cystathionine beta-lyase	*
PADG_07609	Dihydroxy-acid dehydratase	*
PADG_01963	Glycine cleavage system H protein	*
PADG_01286	Iso_dh domain-containing protein	*
PADG_08464	Maleylacetoacetate isomerase	*
PADG_08176	Phosphatidylinositol-phosphatidylcholine	*
	**1.2 Vitamins, cofactors and prosthetic groups metabolism**	
PADG_00282	GTP-binding protein ypt2	1,630245389
PADG_03392	GTP-binding protein YPT52	*
PADG_01626	Ferroxidase	*
PADG_03115	Ran GTPase-binding protein NTF2	*
	**1.3 Carbohydrate metabolism**	
PADG_03514	2-oxoisovalerate dehydrogenase	*
PADG_03984	D-fructose-6-phosphate amidotransferase	*
PADG_00262	Inositol-1-monophosphatase	*
PADG_03943	Phosphomannomutase	*
	**1.4 Nucleotide metabolism and cycle cellular**	
PADG_11958	U6 snRNA-associated Sm-like protein LSm3	*
PADG_02783	RNA-binding La domain-containing protein	*
PADG_03459	RPA_C domain-containing protein	*
PADG_07689	RRM domain-containing protein	*
PADG_01440	ADP/ATP translocase	*
PADG_03669	DNL-type domain-containing protein	*
PADG_01455	KH domain RNA-binding protein	*
PADG_11421	Histone H2A	*
PADG_11347	TIA1 cytotoxic granule-associated RNA binding protein	*
PADG_01852	TPR_REGION domain-containing protein	*
PADG_02346	mRNA stability protein	*
PADG_07515	Protein phosphatase regulator SHP1	*
PADG_01243	Rab GDP dissociation inhibitor	*

PADG_08399	Ras-like protein Rab-11A	*
PADG_03073	Nuclear movement protein nudC	*
	**1.5 Nitrogen and sulfur metabolism**	
PADG_04420	Peptide-methionine (S)-S-oxide reductase	*
	**1.6 Lipid, fatty acid and isoprenoid metabolism**	
PADG_00546	3-oxoacyl-[acyl-carrier protein] reductase	*
PADG_00254	3-oxoacyl-[acyl-carrier-protein] reductase	*
PADG_04774	Dodecenoyl-CoA isomerase	*
PADG_08018	Glycerol-3-phosphate dehydrogenase	*
	**2. Energy**	
	**2. 1 Glycolysis and gluconeogenesis**	
PADG_03099	Aldedh domain-containing protein	*
PADG_07950	Phosphotransferase	*
	**2.2 Pentose-phosphate pathway**	
PADG_03099	Aldedh domain-containing protein	*
	**2.3 Tricarboxylic-acid pathway**	
PADG_08013	Succinate dehydrogenase	*
PADG_01762	Oxoglutarate dehydrogenase	*
	**2.4 Electron transport chain**	
PADG_03785	ATP phosphoribosyltransferase PADG_03785	*
PADG_00688	F-type H+-transporting ATPase subunit H	1,024313448
PADG_05739	NAP family protein	*
	**3. Protein metabolism**	
PADG_07190	Proteasome core particle subunit alpha 2	*
PADG_03965	Proteasome subunit beta	*
PADG_06290	Proteasome alpha 1 domain-containing protein	*
PADG_05160	Dipeptidyl peptidase 3	*
	**4. Protein with binding function or cofactor requirement**	
PADG_00282	GTP-binding protein ypt2	1,630245389
PADG_03392	GTP-binding protein YPT52	*
PADG_01626	Ferroxidase	*
PADG_03115	Ran GTPase-binding protein NTF2	*
	**5. Cell rescue, defense and virulence**	
PADG_07264	Stress-response A/B barrel domain-containing protein	*
PADG_00454	Rhodanese domain-containing protein	*
	**6. Ribossomal metabolism**	
PADG_07583	40S ribosomal protein S21	*
PADG_01914	60S ribosomal protein L35	*
PADG_04612	Ribonucloprotein	*
PADG_03214	Small nuclear ribonucleoprotein	*
PADG_01281	Mitochondrial 37S ribosomal protein MRPS8	*
PADG_04731	KOW domain-containing protein	*
	**7. Translation**	
PADG_05900	Transcription elongation factor 1	*
PADG_08033	Eukaryotic translation initiation	*
PADG_04210	eIF2B_5 domain-containing protein	*

PADG_00878	Isoleucyl-tRNA synthetase	*
PADG_06249	Glutamine--tRNA ligase	*
PADG_04083	Protein-synthesizing GTPase	*
	**8. Unclassified protein**	
PADG_00465	Uncharacterized protein	*
PADG_01380	Uncharacterized protein	*
PADG_02114	Uncharacterized protein	*
PADG_04934	Uncharacterized protein	*
PADG_07506	Uncharacterized protein	1,038740471
PADG_07865	Uncharacterized protein	*
PADG_11424	Uncharacterized protein	*
PADG_01867	Uncharacterized protein	*
PADG_11111	Uncharacterized protein	*

Table S2. ***P. brasiliensis*** **proteins down-regulated after exposure to mebendazole**

Acession number	Protein description	Fold change
	**1. Metabolism**	
	**1.1 Amino acid metabolism**	
PADG_00255	3-hydroxyacyl-[acyl-carrier-protein] dehydratase	-0,38953
PADG_03466	3-hydroxyisobutyrate dehydrogenase	-0,27411
PADG_06876	3-hydroxyisobutyryl-CoA hydrolase	-0,24526
PADG_08468	4-hydroxyphenylpyruvate dioxygenase	0,057708
PADG_08328	5-methyltetrahydropteroyltriglutamate--homocysteine S-methyltransferase	-0,08656
PADG_04374	UTP--glucose-1-phosphate uridylyltransferase	-0,17312
PADG_05492	Serine 3-dehydrogenase	-0,23083
PADG_05111	Serine hydroxymethyltransferase	-0,14427
PADG_05277	Serine hydroxymethyltransferase	-0,33182
PADG_00443	6-hydroxymethyl-7_8-dihydropterin pyrophosphokinase	-0,10099
PADG_01886	Adenosylhomocysteinase	-0,23083
PADG_00002	Alanine ligase	0,014427
PADG_08262	Asparagine synthase	-0,18755
PADG_01621	Aspartate aminotransferase	-0,18755
PADG_03686	Aspartate aminotransferase	0,043281
PADG_04962	Aspartate--tRNA ligase	-0,31739
PADG_01615	Homocitrate synthase	-0,47609
PADG_00663	Homoserine dehydrogenase	-0,23083
PADG_04522	Homoserine kinase	-0,43281
PADG_02719	Methionine adenosyltransferase 2 subunit beta	-0,54822
PADG_02914	Aminomethyltransferase	-0,12984
PADG_03149	Aminopeptidase	-0,2164
PADG_00215	Aromatic-L-amino-acid decarboxylase	-0,04328
PADG_04570	Branched-chain-amino-acid aminotransferase	-0,07213
PADG_03627	2-oxoisovalerate dehydrogenase	-0,28854
PADG_04603	Spermidine synthase	-0,07213
PADG_03522	S-methyl-5'-thioadenosine phosphorylase	-0,23083

PADG_01928	S-adenosylmethionine synthase	-0,17312
PADG_06314	Carboxypeptidase	*
PADG_05058	Chorismate mutase	*
PADG_00888	Citrulline-aspartate ligase	-0,02885
PADG_02456	Cystathionine gamma-lyase CYS3	*
PADG_01418	Cysteine dioxygenase	-0,62036
PADG_06494	Dihydrolipoyl dehydrogenase	-0,1587
PADG_05904	Dihydrolipoyllysine-residue succinyltransferase	-0,12984
PADG_04142	FAA_hydrolase domain-containing protein	*
PADG_08465	Fumarylacetoacetase	-0,11542
PADG_06319	Glutamate decarboxylase	*
PADG_04516	Glutamate dehydrogenase	0,014427
PADG_05337	Glutamate-5-semialdehyde dehydrogenase	*0
PADG_00210	Glycine cleavage system P protein	-0,33182
PADG_07369	Isovaleryl-CoA dehydrogenase	-0,69249
PADG_06429	Ketol-acid reductoisomerase	-0,18755
PADG_08472	Lysine	-0,40395
PADG_05922	M20_dimer domain-containing protein	-0,12984
PADG_07370	Methylcrotonoyl-CoA carboxylase	0
PADG_05085	Multifunctional fusion protein	-0,14427
PADG_08406	O-acetylhomoserine (Thiol)-lyase	-0,17312
PADG_00386	Phospho-2-dehydro-3-deoxyheptonate aldolase	-0,18755
PADG_02123	Aflatoxin B1 aldehyde reductase	*
PADG_07606	D-xylose reductase	0,057708
PADG_01486	Short chain dehydrogenase/reductase family	-0,05771
PADG_06546	Aminopeptidase	0,201977
PADG_02214	4-aminobutyrate aminotransferase	0,115416
PADG_08468	4-hydroxyphenylpyruvate dioxygenase	0,057708
PADG_02751	Acetyl-CoA C-acetyltransferase	0,086562
PADG_00637	Arginase	0,072135
PADG_11833	CN hydrolase domain-containing protein	0,360674
PADG_07782	Deoxyuridine 5'-triphosphate nucleotidohydrolase	0,173123
PADG_06490	Formamidase	0,18755
PADG_08466	Homogentisate 1_2-dioxygenase	0,201977
PADG_02847	HpcH_HpaI domain-containing protein	0,173123
PADG_01328	Ornithine aminotransferase	0,230831
PADG_04657	Nascent polypeptide-associated	0,346247
PADG_00405	Choline dehydrogenase	-0,85119
	**1.2 Vitamins, cofactors and prosthetic groups metabolism**	
PADG_04810	GTP-binding nuclear protein	-0,2164
PADG_08337	GTP-binding protein rhoA	
PADG_08342	GTP-binding protein ypt1	-0,10099
PADG_01530	Guanine nucleotide-binding protein	-0,01443
PADG_01032	Aminoacylproline aminopeptidase	*
PADG_03431	tRNA-binding domain-containing protein	-0,11542
PADG_11950	RanBD1 domain-containing protein	0,274112

PADG_05822	Pyridoxal 5'-phosphate synthase	-0,12984
PADG_07081	Probable electron transfer flavoprotein subunit alpha	0,028854
PADG_06488	PPIase cyclophilin-type domain-containing protein	-0,17312
PADG_04250	Formyltetrahydrofolate synthetase	-0,34625
PADG_00824	HABP4_PAI-RBP1 domain-containing protein	-0,43281
PADG_07749	NAD(P)H:quinone oxidoreductase_ type IV	-0,10099
PADG_05947	Nicotinate-nucleotide pyrophosphorylase	-0,08656
PADG_03176	Phosphatidylglycerol/phosphatidylinositol transfer	-1,15416
PADG_07508	Phosphatidylinositol transfer protein	-0,60593
	**1.3 Carbohydrate metabolism**	
PADG_04718	2-methylcitrate dehydratase	-0,05771
PADG_07699	S-formylglutathione hydrolase	0,490516
PADG_07606	D-xylose reductase	0,057708
PADG_01372	Mannitol-1-phosphate 5-dehydrogenase	0,201977
PADG_07435	Sorbose reductase	-0,11542
	**1.4 Nucleotide metabolism and cycle cellular**	
PADG_04099	5-aminoimidazole-4-carboxamide ribonucleotide formyltransferase	-0,04328
PADG_02683	UV excision repair protein	-0,11542
PADG_02484	Valyl-tRNA synthetase	
PADG_03424	UBA_e1_C domain-containing protein	0,201977
PADG_04242	Sulfhydryl oxidase	0,562651
PADG_00422	RRM domain-containing protein	0,129843
PADG_05893	Histone chaperone NAP1	-0,12984
PADG_05907	Histone H2B	0,346247
PADG_06768	Histone-glutamine methyltransferase	0,072135
PADG_05906	Histone H2A	-0,2164
PADG_02246	Adenosine kinase	0,360674
PADG_00832	Adenylosuccinate synthetase	0,562651
PADG_00873	Histone H3	-0,07213
PADG_00872	Histone H4	0
PADG_07134	Histone H4	-0,04328
PADG_01565	Calnexin	
PADG_02899	U2 snRNP complex subunit CUS2	
PADG_00144	U6 snRNA-associated Sm-like protein LSm2	
PADG_05897	Seryl-tRNA synthetase	
PADG_00001	Peptidyl-prolyl cis-trans isomerase	0,057708
PADG_02555	Nucleic acid-binding protein	0,100989
PADG_01711	Hsp90 co-chaperone AHA1	0,18755
PADG_04866	S10_plectin domain-containing protein	-0,18755
PADG_05034	RRM domain-containing protein	-0,14427
PADG_11711	RNA helicase	-0,41838
PADG_01151	RNA_pol_L_2 domain-containing protein	
PADG_08724	RPEL repeat protein	-0,04328
PADG_04912	Perodoxin	
PADG_03841	Protein disulfide-isomerase	-0,04328
PADG_04034	Chaperone Dna	-0,10099

PADG_07023	Carn_acyltransf domain-containing protein	-0,28854
PADG_08048	CCT-beta	-0,80791
PADG_05683	Cell division control protein	-0,36067
PADG_05139	Chaperone protein dnaK	-0,02885
PADG_02763	Cyclin-dependent kinases regulatory	-0,17312
PADG_05848	Diadenosine tetraphosphate synthetase	-0,50494
PADG_02845	Diploid state maintenance protein chpA	0,043281
PADG_05837	E3 ubiquitin ligase complex SCF subunit	-0,05771
PADG_03562	Endoplasmic reticulum chaperone	-0,02885
PADG_02652	Grp1p	-0,05771
PADG_06992	GrpE protein homolog	0,014427
PADG_05183	Grx4 family monothiol glutaredoxin	-1,54368
PADG_07585	Inosine-5'-monophosphate dehydrogenase	-0,1587
PADG_00207	J domain-containing protein	-0,1587
PADG_04899	Lactamase	
PADG_04288	L-PSP endoribonuclease family protein (Hmf1)	-0,24526
PADG_11857	Mitotic checkpoint protein BUB3	
PADG_00656	Non-histone chromosomal protein 6	-0,2164
PADG_00849	Nuclear segregation protein Bfr1	-0,66364
PADG_07524	Nucleoside diphosphate kinase	-0,08656
PADG_02183	Nudix hydrolase domain-containing protein	
PADG_05474	Nudix hydrolase domain-containing protein	
PADG_05225	Orotidine 5'-phosphate decarboxylase	-0,89447
PADG_00111	Oxidative DNA demethylase	-0,47609
	**1.5 Nitrogen and sulfur metabolism**	
	Urease PADG_07010	-0,3751
	Uricase PADG_00331	-0,14427
	Carbonic anhydrase PADG_07674	
	Nitroreductase domain-containing protein PADG_02048	-0,17312
	**1.6 Lipid, fatty acid and isoprenoid metabolism**	
PADG_03194	3-ketoacyl-CoA thiolase B	-0,25969
PADG_01687	3-ketoacyl-coA thiolase	-0,23083
PADG_06805	Acyl-CoA dehydrogenase	-0,02885
PADG_01209	Enoyl-CoA hydratase	-0,11542
PADG_12025	Glutaryl-CoA dehydrogenase	0
PADG_06382	Acetyl-CoA acetyltransferase	-0,08656
PADG_04687	3-beta-hydroxysteroid dehydrogenase	0,158696
PADG_01564	Methylmalonate-semialdehyde dehydrogenase	-0,1587
	**1.7 Secondary metabolism**	
PADG_04636	DLH domain-containing protein	-0,11542
	**2. Energy**	
	**2. 1 Glycolysis and gluconeogenesis**	
PADG_04059	Enolase	-0,11542
PADG_07213	Acetyltransferase	-0,04328
PADG_03403	Aldedhyde dehydrogenase domain-containing protein	-0,17312
PADG_06740	Aldedhyde dehydrogenase domain-containing protein	-0,08656

PADG_05081	Aldehyde dehydrogenase	0,057708
PADG_02411	Glyceraldehyde-3-phosphate dehydrogenase	-0,01443
PADG_01706	Fructose-bisphosphatase	-0,2164
PADG_00668	Fructose-bisphosphate aldolase	-0,23083
PADG_04103	Pyruvate carboxylase	-0,31739
PADG_07907	Pyruvate decarboxylase	-0,27411
PADG_00714	Pyruvate decarboxylase	-0,04328
PADG_02063	Pyruvate dehydrogenase E1 component alpha subunit	-0,18755
PADG_00246	Pyruvate dehydrogenase E1 component subunit beta	-0,07213
PADG_01278	Pyruvate kinase	-0,14427
PADG_02145	Alpha-1_4 glucan phosphorylase	-0,04328
PADG_03276	S-(hydroxymethyl)glutathione dehydrogenase	0
PADG_00451	Glucose-6-phosphate isomerase	-0,01443
PADG_11132	Phosphoglucomutase	-0,04328
PADG_01896	Phosphoglycerate kinase	-0,05771
PADG_05109	Phosphoglycerate mutase	-0,11542
PADG_05081	Aldehyde dehydrogenase	0,057708
PADG_06906	Triosephosphate isomerase	0,158696
PADG_03118	Glucose-6-phosphate 1-epimerase	0,129843
PADG_02981	DJ-1_PfpI domain-containing protein	-0,20198
	**2.2 Pentose-phosphate pathway**	
PADG_06054	2-deoxy-D-ribose 5-phosphate aldolase	
PADG_03651	6-phosphogluconate dehydrogenase_ decarboxylating	-0,23083
PADG_04604	Transketolase	0,043281
PADG_07420	Transaldolase	-0,02885
PADG_03859	Chlorophyll synthesis pathway protein	-0,17312
	**2.3 Tricarboxylic-acid pathway**	
PADG_11845	Aconitate hydratase	0
PADG_04993	ATP citrate synthase	-0,28854
PADG_04994	ATP citrate synthase	-0,79348
PADG_02561	ATP synthase subunit alpha	-0,2164
PADG_04729	ATP synthase subunit d_ mitochondrial	-0,14427
PADG_07813	ATP synthase subunit gamma	-0,41838
PADG_07210	Malate dehydrogenase	-0,04328
PADG_08054	Malate dehydrogenase	-0,10099
PADG_04702	Malate synthase	-0,38953
PADG_00052	Succinate dehydrogenase	-0,80791
PADG_00317	Succinate--CoA ligase	
PADG_02260	Succinate--CoA ligase	0,043281
PADG_03058	Succinate-semialdehyde dehydrogenase	-0,54822
PADG_04939	Succinyl-CoA	-0,12984
PADG_02728	Sulfite oxidase	-0,12984
PADG_04710	Citrate synthase	-0,12984
PADG_08387	Citrate synthase	0,028854
PADG_06221	Formate dehydrogenase	-0,64921
PADG_08119	Fumarate hydratase	-0,02885

PADG_02805	Isocitrate dehydrogenase [NAD] subunit_ mitochondrial	-0,46166
PADG_03977	Isocitrate dehydrogenase [NAD] subunit_ mitochondrial	-0,34625
PADG_04249	Isocitrate dehydrogenase	-0,1587
PADG_01483	Isocitrate lyase	-0,04328
PADG_00171	L-lactate dehydrogenase (cytochrome)	-0,11542
PADG_08503	Phosphoenolpyruvate carboxykinase	-0,18755
	**2.4 Electron transport chain**	
PADG_06956	Vacuolar proton pump	
PADG_02592	Cytochrome b5	-0,01443
PADG_04559	Cytochrome b5	-0,23083
PADG_06978	Cytochrome c	
PADG_05750	Cytochrome c oxidase	-0,24526
PADG_08349	ATP synthase subunit beta	0,072135
PADG_00192	ATP-dependent 6-phosphofructokinase	0,375101
PADG_07964	H(+)-transporting V1 sector ATPase	-0,7502
PADG_04175	Inorganic diphosphatase	-0,01443
PADG_06196	Oxidored_FMN domain-containing protein	-0,02885
PADG_00366	NAD(P)-bd_dom domain-containing protein	0,504943
PADG_05523	PKS_ER domain-containing protein	-0,23083
PADG_07836	PKS_ER domain-containing protein	-0,04328
	**2.5 Fermention**	
PADG_11405	Alcohol dehydrogenase	-0,44724
	**3. Protein metabolism**	
PADG_07190	Proteasome core particle subunit alpha 2	
PADG_02735	Proteasome subunit alpha type	0,259685
PADG_03965	Proteasome subunit beta	
PADG_02735	Proteasome subunit alpha type	0,259685
PADG_06290	Proteasome alpha_domain-containing protein	
PADG_05160	Dipeptidyl peptidase	
	**4. Protein with binding function or cofactor requirement**	
PADG_04810	GTP-binding nuclear protein	-0,2164
PADG_08337	GTP-binding protein rhoA	
PADG_08342	GTP-binding protein ypt1	-0,10099
PADG_01530	Guanine nucleotide-binding protein	-0,01443
PADG_01032	Aminoacylproline aminopeptidase	
PADG_03431	tRNA-binding domain-containing protein	-0,11542
PADG_05822	Pyridoxal 5'-phosphate synthase	-0,12984
PADG_07081	Probable electron transfer flavoprotein subunit alpha	0,028854
PADG_06488	PPIase cyclophilin-type domain-containing protein	-0,17312
PADG_04250	Formyltetrahydrofolate synthetase	-0,34625
PADG_00824	HABP4_PAI-RBP1 domain-containing protein	-0,43281
PADG_07749	NAD(P)H:quinone oxidoreductase_ type IV	-0,10099
PADG_11950	RanBD1 domain-containing protein	0,274112
PADG_05947	Nicotinate-nucleotide pyrophosphorylase	-0,08656
PADG_03176	Phosphatidylglycerol/phosphatidylinositol transfer	-1,15416
PADG_07508	Phosphatidylinositol transfer protein	-0,60593

	**5. Cell rescue, defense and virulence**	
PADG_08369	Hsp60-like protein	-0,17312
PADG_08118	Hsp72-like protein	-0,04328
PADG_02761	Hsp75-like protein	-0,01443
PADG_00430	Hsp7-like protein	-0,12984
PADG_02030	Hsp90 chaperone protein kinase	-0,11542
PADG_04379	Hsp90 cochaperone STI1	-0,08656
PADG_07715	Hsp90-like protein	-0,23083
PADG_02785	Heat shock protein Hsp88	-0,05771
PADG_01363	ACB domain-containing protein	-0,07213
PADG_07627	4-carboxymuconolactone decarboxylase	0
PADG_05032	Hsp90 cochaperone SBA1	-0,30297
PADG_01755	Superoxide dismutase	-0,2164
PADG_07418	Superoxide dismutase	-0,11542
PADG_03095	Thioredoxin domain-containing protein	0,028854
PADG_07946	Thioredoxin domain-containing protein	-0,18755
PADG_05504	Thioredoxin	-0,07213
PADG_01551	Thioredoxin reductase	-0,17312
PADG_03163	Peroxidase	-0,01443
PADG_03963	30 kDa heat shock protein	0,115416
PADG_00446	Oxidoreductase 2-nitropropane dioxygenase	-0,18755
	**6. Ribossomal metabolism**	
PADG_00044	Ribonucleo protein	-0,34625
PADG_01407	40S ribosomal protein	-0,27411
PADG_07685	40S ribosomal protein	0,028854
PADG_06525	40S ribosomal protein S1	-0,33182
PADG_01267	40S ribosomal protein S11	-0,14427
PADG_01427	40S ribosomal protein S12	-0,04328
PADG_00335	40S ribosomal protein S14	-0,24526
PADG_00354	40S ribosomal protein S17	0
PADG_06313	40S ribosomal protein S18	-0,1587
PADG_12324	40S ribosomal protein S19	-0,3751
PADG_08602	40S ribosomal protein S2	-0,12984
PADG_06502	40S ribosomal protein S20	-0,23083
PADG_04315	40S ribosomal protein S24	-0,43281
PADG_06599	40S ribosomal protein S25	
PADG_08238	40S ribosomal protein S26	-0,24526
PADG_06048	40S ribosomal protein S27	-0,23083
PADG_08605	40S ribosomal protein S28	-0,14427
PADG_03315	40S ribosomal protein S4	-0,10099
PADG_06838	40S ribosomal protein S5	-0,25969
PADG_01654	40S ribosomal protein S6	0,043281
PADG_00942	40S ribosomal protein S7	-0,30297
PADG_03326	40S ribosomal protein S9	-0,01443
PADG_02446	60S acidic ribosomal protein P2	-0,3751
PADG_04030	60S acidic ribosomal protein	-0,57708

PADG_08244	60S acidic ribosomal protein	-0,24526
PADG_00612	60S ribosomal protein	-0,24526
PADG_04106	60S ribosomal protein L11	-0,10099
PADG_07803	60S ribosomal protein L12	-0,40395
PADG_11227	60S ribosomal protein L13	-0,25969
PADG_05338	60S ribosomal protein L18-B	-0,18755
PADG_02249	60S ribosomal protein L2	-0,18755
PADG_03873	60S ribosomal protein L20	-0,18755
PADG_03325	60S ribosomal protein L21	-0,27411
PADG_04449	60S ribosomal protein L23	-0,38953
PADG_05883	60S ribosomal protein L25	0,014427
PADG_12253	60S ribosomal protein L3	-0,23083
PADG_11832	60S ribosomal protein L31	-0,40395
PADG_01083	60S ribosomal protein L32	
PADG_04402	60S ribosomal protein L34	-0,23083
PADG_04065	60S ribosomal protein L36	-0,11542
PADG_05836	60S ribosomal protein L39	-0,12984
PADG_01026	60S ribosomal protein L43	-0,01443
PADG_05244	60S ribosomal protein L44	-0,1587
PADG_11379	60S ribosomal protein L5	-0,28854
PADG_02888	60S ribosomal protein L6	-0,20198
PADG_01387	60S ribosomal protein L7	-0,18755
PADG_04848	60S ribosomal protein L8	-0,24526
PADG_05025	Ribosomal protein L24	-0,1587
PADG_02056	Ribosomal protein L7/L12	-0,24526
PADG_02828	Ribosomal protein	-0,34625
PADG_08715	Ribosomal_L28e domain-containing protein	-0,07213
PADG_02445	40S ribosomal protein	0,346247
PADG_00784	40S ribosomal protein S0	0,706921
PADG_00333	40S ribosomal protein S16	0,100989
PADG_07870	Ribosomal_S7 domain-containing protein	-0,04328
PADG_12365	40S ribosomal protein S8	0,086562
PADG_03778	60S ribosomal protein L10-A	0,072135
PADG_00514	60S ribosomal protein L16	0,129843
PADG_04588	60S ribosomal protein L22	0,129843
PADG_05939	60S ribosomal protein L27	0,288539
PADG_03781	60S ribosomal protein L30	0,086562
PADG_07834	Ubiquitin carboxyl-terminal hydrolase	0,043281
PADG_00607	Riboflavin synthase	0,533797
PADG_06856	Small nuclear ribonucleoprotein	0,072135
PADG_07891	Ubiquitin	-0,24526
PADG_04612	Ribosomal protein L15	0,086562
PADG_02637	E2 ubiquitin-conjugating enzyme	0,302966
PADG_00995	Ubiquitin-40S ribosomal protein	-0,24526
PADG_05721	Ribos_L4_asso_C domain-containing protein	-0,1587
	**7. Transport routes**	

PADG_04440	14_3_3 domain-containing protein	-0,40395
PADG_04056	14-3-3 family protein	-0,20198
PADG_07014	Vesicular-fusion protein	-0,14427
PADG_02833	ADP-ribosylation factor	0,028854
PADG_02017	Calmodulin	-0,10099
PADG_06515	Suaprga1	-0,30297
PADG_05517	Rho GDP-dissociation inhibitor	0,043281
	**8. Biogenesis of cellular components**	
PADG_03449	Isopentenyl-diphosphate Delta-isomerase	0,129843
PADG_05356	Isochorismatase domain-containing protein	0,548224
PADG_03449	Isopentenyl-diphosphate Delta-isomerase	0,129843
PADG_06165	Glycolipid transferprotein HET-C2	0,764628
	**9. Translation**	
PADG_01949	Elongation factor	-0,17312
PADG_06265	Elongation factor 1 gamma	-0,23083
PADG_00692	Elongation factor 1-alpha	-0,40395
PADG_08125	Elongation factor 2	-0,27411
PADG_06110	Translation machinery-associated protein	-0,36067
PADG_06568	Translationally-controlled tumor protein homolog	-0,05771
PADG_07884	Polyadenylate-binding protein	-0,31739
PADG_02896	EF1_GNE domain-containing protein	-0,14427
PADG_04251	Eukaryotic translation initiation	
PADG_02691	Eukaryotic translation initiation	-0,1587
PADG_01558	Histidine--tRNA ligase	-0,99546
PADG_00001	Peptidyl-prolyl cis-trans isomerase	0,057708
PADG_04092	Peptidyl-prolyl cis-trans isomerase	0,043281
PADG_07953	Peptidyl-prolyl cis-trans isomerase	0,028854
PADG_12323	Peptidyl-prolyl cis-trans isomerase	-0,04328
PADG_01406	Phenylalanyl-tRNA synthetase beta subunit	-0,02885
	**10. Unclassified protein**	
PADG_02343	Uncharacterized protein	-0,1587
PADG_04311	Uncharacterized protein	-0,46166
PADG_04343	Uncharacterized protein	-0,12984
PADG_04446	Uncharacterized protein	
PADG_05798	Uncharacterized protein	-0,01443
PADG_00541	Uncharacterized protein	-0,62036
PADG_00608	Uncharacterized protein	-0,41838
PADG_00921	Uncharacterized protein	-0,1587
PADG_01773	Uncharacterized protein	
PADG_01796	Uncharacterized protein	
PADG_01871	Uncharacterized protein	
PADG_02764	Uncharacterized protein	-0,08656
PADG_03005	Uncharacterized protein	
PADG_03660	Uncharacterized protein	-0,17312
PADG_04439	Uncharacterized protein	-0,04328
PADG_06136	Uncharacterized protein	-0,5338

PADG_06289	Uncharacterized protein	-0,04328
PADG_07422	Uncharacterized protein	-0,12984
PADG_07714	Uncharacterized protein	-0,60593
PADG_11062	Uncharacterized protein	-0,30297
PADG_01052	Uncharacterized protein	
PADG_00322	Uncharacterized protein	-0,31739
PADG_12447	Uncharacterized protein	-0,04328
PADG_02967	Uncharacterized protein	-0,1587
PADG_00240	Uncharacterized protein	0,822336
PADG_01857	Uncharacterized protein	0,403955
PADG_02092	Uncharacterized protein	0,100989
PADG_03671	Uncharacterized protein	0,389528
PADG_08212	Uncharacterized protein	0,389528
PADG_08569	Uncharacterized protein	0,129843
PADG_04869	Uncharacterized protein	0,14427

Disclaimer/Publisher's Note: The statements, opinions and data contained in all publications are solely those of the individual author(s) and contributor(s) and not of MDPI and/or the editor(s). MDPI and/or the editor(s) disclaim responsibility for any injury to people or property resulting from any ideas, methods, instructions or products referred to in the content.
